# Modelling Human Regulatory Variation in Mouse: Finding the Function in Genome-Wide Association Studies and Whole-Genome Sequencing

**DOI:** 10.1371/journal.pgen.1002544

**Published:** 2012-03-01

**Authors:** Jean-François Schmouth, Russell J. Bonaguro, Ximena Corso-Diaz, Elizabeth M. Simpson

**Affiliations:** 1 Centre for Molecular Medicine and Therapeutics at the Child and Family Research Institute, University of British Columbia, Vancouver, Canada; 2 Genetics Graduate Program, University of British Columbia, Vancouver, Canada; 3 Department of Medical Genetics, University of British Columbia, Vancouver, Canada; 4 Department of Psychiatry, University of British Columbia, Vancouver, Canada; Harvard Medical School, United States of America

## Abstract

An increasing body of literature from genome-wide association studies and human whole-genome sequencing highlights the identification of large numbers of candidate regulatory variants of potential therapeutic interest in numerous diseases. Our relatively poor understanding of the functions of non-coding genomic sequence, and the slow and laborious process of experimental validation of the functional significance of human regulatory variants, limits our ability to fully benefit from this information in our efforts to comprehend human disease. Humanized mouse models (HuMMs), in which human genes are introduced into the mouse, suggest an approach to this problem. In the past, HuMMs have been used successfully to study human disease variants; e.g., the complex genetic condition arising from Down syndrome, common monogenic disorders such as Huntington disease and β-thalassemia, and cancer susceptibility genes such as *BRCA1*. In this commentary, we highlight a novel method for high-throughput single-copy site-specific generation of HuMMs entitled High-throughput Human Genes on the X Chromosome (HuGX). This method can be applied to most human genes for which a bacterial artificial chromosome (BAC) construct can be derived and a mouse-null allele exists. This strategy comprises (1) the use of recombineering technology to create a human variant–harbouring BAC, (2) knock-in of this BAC into the mouse genome using *Hprt* docking technology, and (3) allele comparison by interspecies complementation. We demonstrate the throughput of the HuGX method by generating a series of seven different alleles for the human *NR2E1* gene at *Hprt*. In future challenges, we consider the current limitations of experimental approaches and call for a concerted effort by the genetics community, for both human and mouse, to solve the challenge of the functional analysis of human regulatory variation.

## Introduction

**Figure 1:**
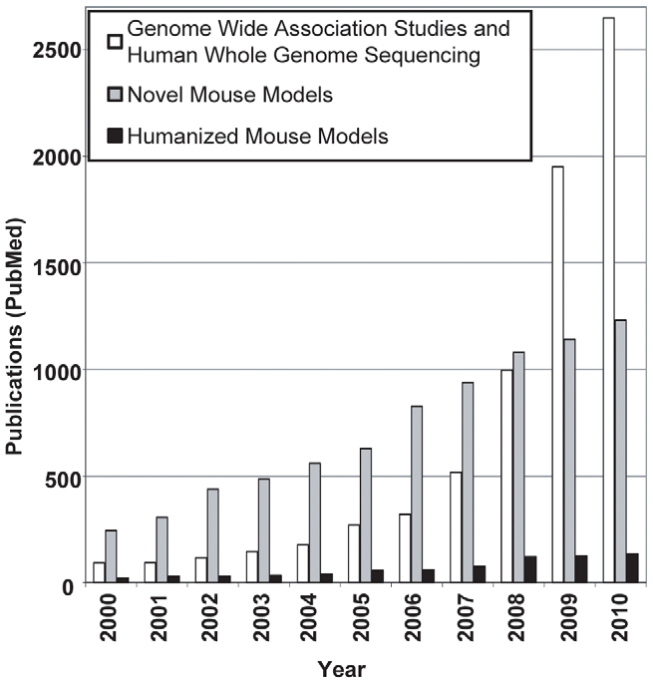
The literature is increasing more slowly for humanized mouse models than for GWASs and HWGS or novel mouse models. Interrogation of the PubMed literature database (http://www.ncbi.nlm.nih.gov/pubmed) reveals a faster growing body of literature related to GWASs and HWGS (white bars) or novel mouse models (grey bars) than to HuMMs (black bars). Interrogation of the database was done using the online search option from EndNote (http://www.endnote.com/). Individual numbers of entries for the search terms “genome wide association studies” and “human whole genome sequencing” were added together for the figure. Search terms for novel mouse models were “novel knockout mouse”, “novel knockin mouse”, and “novel knock-in mouse”. The entries for the search term “humanized mouse models” were not restricted to genetic mouse models but included xenograft mouse models as well. Search terms were interrogated in “all fields” per year.

A decade ago, the Human Genome Project published its first human DNA sequence draft, followed shortly by the full version in 2003 [1]–[3]. This project and the SNP Consortium and the International HapMap Project have provided geneticists with invaluable tools for their research on human populations [4], [5]. Their activities have resulted in an exponential growth of PubMed entries related to genome-wide association studies (GWASs) plus human whole-genome sequencing (HWGS) over the past decade (Figure 1, white bars). The increasing numbers of studies cumulated at 2,649 entries in 2010; these studies mainly focused on understanding the genetic variants affecting the development of diseases and disorders in humans. For obvious reasons, protein-coding variants have been the most extensively studied so far. However, an increasing body of literature from GWASs and candidate gene association studies also highlights the identification of candidate regulatory variants of potential therapeutic interest in numerous diseases [6]–[14]. Furthermore, with the cost of HWGS being driven down by cheaper sequencing technologies, we envision a continuing large increase in the identification of candidate regulatory variants. In general, the biological role of variants found in putative regulatory regions is harder to predict than that for protein-coding variants, in part because of our poor understanding of the functions of non-coding genomic sequence, and the slow and laborious process of experimental validation of the functional significance of human regulatory variants. In this commentary, we will review current efforts at modelling human variation in mouse and highlight a novel method for high-throughput generation of humanized mouse models (HuMMs) entitled High-throughput Human Genes on the X Chromosome (HuGX, pronounced “hugs”).

## Typical Humanized Mouse Models Are Powerful but Not Ideal for Regulatory Variants

It is always important to remember that mice are not “little humans”, and that species-specific differences limit the value of any model organism. Nevertheless, throughout history, the laboratory mouse has been the human disease model of choice for geneticists, in part because of the rapid breeding rate of mice, which led to the generation of a wide variety of inbred and spontaneous-mutation-harbouring strains. Contributing to the mouse as a model was the advancement in embryonic technologies, allowing the engineering of the mouse genome and resulting in the generation of transgenic random-insertion, knock-out, and knock-in mouse models. Furthermore, the laboratory mouse genome sequence was released in 2002 and demonstrated that 99% of mouse genes have human homologues, strengthening the importance of mouse models in probing human biology and disease [15]–[17]. This importance has been reflected by a continually growing literature describing novel mouse models over the past decade (Figure 1, grey bars). However, in contrast to coding regions, human–mouse comparative genomic analysis demonstrated a lower level of conservation in putative regulatory regions of the genome [15]. This finding strengthened a hypothesis posed more than 25 years ago suggesting that regulatory regions may play a crucial role in underlying species differences and human-specific biology and disease [18]. It also raises a problem for mouse modelling when a strictly mouse-genome-based approach is used to validate human candidate regulatory variants, since the equivalent DNA sequence and/or epigenomic environment may not be present.

HuMMs, in which human genes are introduced into the mouse, suggest an approach to this problem. Surprisingly, the number of entries in the literature for HuMMs is very modest when compared to the two previous categories (Figure 1, black bars). Many of the HuMM entries are not genetic per se but are related to immunity studies—using human cells or tissues engrafted in nude mice—and thus are unrelated to the data generated by GWASs and HWGS. Nevertheless, there are numerous examples of successful genetic HuMMs.

A HuMM approach was used to study the complex genetic condition arising from Down syndrome, also known as trisomy 21. This syndrome results from an altered dosage of wild-type (WT) genes on human Chromosome 21, a phenomenon that can be mimicked by generating trans-species aneuploid mice carrying a human chromosome [19]. In this example, the mouse strain generated contained an estimated 92% of all known human Chromosome 21 genes, and a large-scale analysis demonstrated that 81% of human Chromosome 21 genes were expressed in mouse tissues [19], [20]. Additional investigation, using a set of conserved and well-characterized transcription factors responsible for hepatocyte development and function, revealed that genetic sequence rather than interspecies differences in epigenetic machinery or cellular environment is largely responsible for directing transcriptional programs [21]. These results demonstrated that human gene regulation is generally conserved in mice, strengthening the argument that HuMMs can be a good approach for understanding the role of candidate regulatory variants in disease development.

Other examples of successful HuMMs to study the role of genetic mutations are found in common monogenic disorders such as Huntington disease and β-thalassemia, as well as cancer susceptibility genes such as *BRCA1* [22]–[25]. All of these WT human genes in HuMMs successfully rescued the embryonic lethal phenotype from the mouse gene knock-out animals, thereby providing valuable information regarding the human gene function by demonstrating an interspecies complementation of the human gene in the mouse null background. This complementation was due not only to the similarity of the genes in terms of protein function, but also to the identical tissue expression distribution of the human gene [22]–[25]. This was surprising considering the low percentage of identity between human and mouse for some of these genes in both the regulatory and coding sequences [25].

These results were invaluable, as they demonstrated that HuMMs can be used to study the biological role of mutant forms of these human genes. In the case of Huntington disease, this line of investigation has led to the generation of several human yeast artificial chromosome (YAC)–harbouring strains to study the biological implication of expanded glutamine repeats in Huntington disease development [26]–[28]. Advancements in site-specific bacterial artificial chromosome (BAC) mutagenesis techniques supported the shift to generation of BAC-based mutation-harbouring mouse models [29]–[32]. These included the generation of HuMMs harbouring codon-specific mutations for β-thalassemia and the *BRCA1* cancer susceptibility gene. These HuMMs provided information regarding the biological implication of such mutations and their potential underlying role in human health [33], [34]. However, the approaches used to generate these HuMMs, which were suitable when protein-coding variants were being tested, encountered serious limitations in probing the role of human candidate regulatory variants.

In general, HuMM generation has used microinjection of DNA into zygotic pronuclei [35]–[37]. This technique is widely used in the field of mammalian genetics, but is not without limitations. For one, it requires extensive characterization of the different founder lines to control for variability in gene expression, a phenomenon due in part to the influence of the genomic environment at the site of insertion (i.e., position effect) and the number of copies often found tandemly inserted (i.e., copy effect) [38]–[41]. The transgene can potentially lead to disruption of endogenous gene function and repeat-induced gene silencing, two factors that must be taken into account when generating mice by random-insertion pronuclear injection [42]. Since each strain is unique, reproducibility between the different mouse strains becomes a major limiting factor when using random insertion as a mean to generate HuMMs. This lack of reproducibility is less than ideal for any comparison between transgenes in different mouse strains, but is particularly concerning when probing for candidate regulatory variant differences. The ideal method would control for both the site of insertion and the copy numbers inserted in the genome.

## Excellent Techniques Exist for Single-Copy Non-Random Docking in the Mouse Genome

One type of approach, which allows single-copy insertion in the genome, includes the use of retroviruses and transposon activity [43]–[45]. Although quite successful, this approach has limitations as it does not provide controls for the site of insertion in the genome, leading to variability in expression due to genomic environment, as well as potential disruption of endogenous genes. Another potentially powerful approach, called recombinase-mediated genomic replacement, allows the cre-based insertion of a human gene at the site of, and replacing, the endogenous mouse gene [46]. This approach provides stringent control over the genomic environment surrounding the insertion site. However, the method simultaneously creates two inseparable genetic events in the same gene: (1) heterozygosity at the mouse locus and (2) addition of the human gene. Thus, the human gene can be studied only on the heterozygous mutant mouse background. Other limitations include the fact that the replacement is a low-frequency event, and the “gene by gene” approach will restrict throughput. Another novel approach was described recently using pronuclear injection coupled to integrase activity to achieve single-copy site-specific insertion in the mouse genome [47]. This approach used φC31-integrase-mediated recombination activity between *attB* sites from recombinant DNA with *attP* sites previously inserted at a specific locus in the mouse genome. Although also quite promising, this approach yielded up to 40% site-specific integration at best, and was only tested on small construct plasmids, another limitation, since many genes require large constructs [47].

Traditionally, two mouse genes have been used as genomic docking sites: the autosomal *Rosa26* (*reverse orientation splice acceptor 26*) and X chromosome *Hprt* (*hypoxanthine guanine phosphoribosyl transferase*) [48], [49]. The *Rosa26* locus has most often been used to dock constructs when strong ubiquitous expression is required [50]–[54]. Plasmid-size docking is readily achieved; however, large BAC insertions have not been reported. Also, insertion at the *Rosa26* locus typically results in disruption of the gene, which in turn may lead to mild phenotypic consequences [55]. Use of the *Hprt* docking site has also been widely reported in the literature, and despite the wide expression of *Hprt* itself, this locus is more often chosen for tissue- or cell-type-specific expression of the targeted construct [56]–[58]. This locus readily accepts plasmid-size constructs but also large (>200 kb) BAC constructs [48], [56]. In the past, docking has been done in such a way that it disrupts the *Hprt* gene, resulting in mice with a mild phenotype [59]–[61]. However, this disruption is now typically avoided by a strategy that uses embryonic stem cells (ESCs) that already carry a spontaneous deletion of the 5′ end of the *Hprt* gene [62]. In this strategy, docking involves construct insertion 5′ of *Hprt* and repairing the expression of the *Hprt* gene itself [56], [57], [63]. This repair of *Hprt* enables direct selection of high-frequency correctly targeted ESC clones [63].

## GWASs and HWGS Require High-Throughput Humanized Mouse Models

Huge strides have been made bringing high throughput to mouse functional genomics. One such stride is simple and highly efficient BAC recombineering in *Escherichia coli* [31], [64], [65]. This technology provides researchers with limitless possibilities for DNA modification via homologous recombination in *E. coli*. It employs the BAC-adapted strain harbouring a defective lambda prophage that allows the recombination genes *exo*, *bet*, and *gam* to be expressed under the control of a temperature-sensitive λ cI-repressor [31], [64]–[66]. DNA modification possibilities include insertion of exogenous DNA fragments in the endogenous BAC DNA, size-specific DNA deletion, single-base-pair-specific DNA alteration, and BAC fusion (i.e., recombining overlapping BAC constructs into a single, larger BAC) [30], [31], [66], [67]. Hence, generation of any variant-harbouring allele in a high-throughput manner can be easily achieved using this technology. Such approaches are already being adopted by large-scale mouse knock-out programmes such as the International Knockout Mouse Consortium [68].

Another stride is the generation of important resources by the currently ongoing large-scale mouse projects [58], [68], [69]. For example, the International Knockout Mouse Consortium is generating ESC-targeted mutations in all protein-coding genes [68]. This resource will have many impacts, but specific to this discussion, it enables complementation approaches to be undertaken for most human genes. To date, this group has generated 16,878 targeted alleles in germline-competent C57BL/6N ESCs (http://www.knockoutmouse.org) [68], [70]–[72].

Finally, C57BL/6 is the most widely used inbred mouse strain and one of the best characterized [68]. The increasing use of ESCs derived from this strain, especially by large-scale projects, will greatly reduce the need for backcrossing by projects using this mouse strain, thus increasing the throughput of most projects [68].

Even with these game-changing strides, HuMM generation will never achieve the throughput of array and sequence technologies. Thus, variants identified by GWASs and HWGS will always need to be extensively pre-screened as strong candidate regulatory variants and suitable for cross-species analysis before entering a HuMM project pipeline.

## HuGX for High-Throughput Assaying of Human Candidate Regulatory Variants

Here we present an approach, HuGX, aimed at understanding the role of candidate human regulatory variants in the development of human diseases and disorders. The strategy comprises (1) the use of the BAC-adapted recombineering technology to create a human-variant-harbouring BAC, (2) knock-in of this BAC into the mouse genome using *Hprt* docking technology, and (3) allele comparison by interspecies complementation. This approach can be applied to human genes for which an expressing BAC construct can be derived, which can complement at least a component of a mouse phenotype.

**Figure 2:**
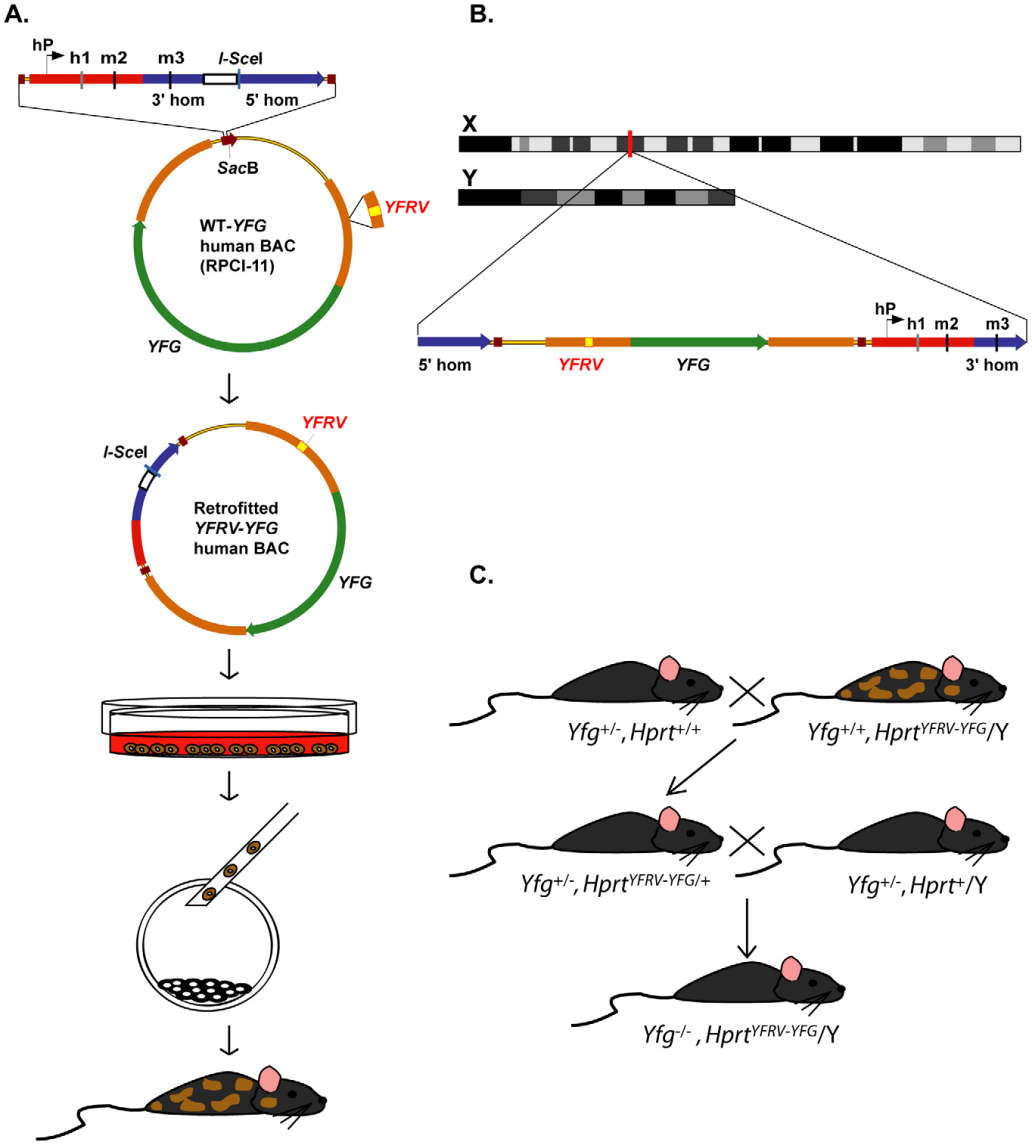
Strategy for high-throughput human genes on the X chromosome (HuGX). (A) Flow diagram representing the major steps of the HuGX strategy, which builds on previous methods [56], [74]. Starting with a human BAC carrying your favourite gene (*YFG*) from the RPCI-11 library, for example, two retrofitting steps are employed: (1) addition of the *HPRT* homology regions for recombination (WT-*YFG*) and (2) introduction of your favourite regulatory variant (*YFRV*) into *YFG* (*YFRV-YFG*). In this example the resulting BAC *YFRV*-*YFG* is linearized, typically using I-SceI, and electroporated into ESCs. 129P2/OlaHsd, B6129F1 hybrid, and C57BL/6NTac ESCs are all available carrying the 36-kb (*Hprt^b-m3^*) deletion used for docking. Selection of homologous recombinant clones is performed using hypoxanthine-aminopterin-thymidine medium, and clones carrying correctly targeted complete-BAC inserts are injected into blastocysts to generate chimeras. Schematic, not to scale. (B) Details of knock-in 5′ of the *Hprt* locus on X chromosome. The linearized BAC construct is introduced into the *Hprt^b-m3^* deletion by electroporation. *Hprt* gene expression is restored by the presence of the human *HPRT* promoter (hP), first exon (h1), and second mouse exon (m2). Mouse homology arms (blue); *Hprt* coding regions (red); vector backbone (narrow yellow line); *Sac*B gene from BAC vector backbone (brown); 5′ and 3′ untranslated regions of *YFG* (orange); *YFRV* (yellow); coding region of *YFG* (green); hP (black arrow); h1 (grey); m2 and m3 (black). Schematic, not to scale. (C) Breeding strategy to achieve complementation. Assuming the genetic background is suitable, male chimeras can immediately be bred to females heterozygous for a null allele at the mouse copy of *Yfg* (*Yfg* ^+/−^) to generate germline females heterozygous for *Yfg* (*Yfg* ^+/−^) and *Hprt^YFRV-YFG^* ^/+^. On the other hand, assuming the challenging situation in which no heterozygous phenotype exists to complement, these females will be mated with a *Yfg* ^+/−^male, resulting in males for study carrying a single copy of the human retrofitted *Hprt^YFRV-YFG^* and the mouse null (*Yfg* ^−/−^) gene.

The first step is to find a suitable BAC for “your favourite gene” (*YFG*). This BAC should be computationally analyzed to determine the likelihood that it includes the entire coding sequence as well as 5′ and 3′ regulatory sequences. The GENSAT project, having generated >1,000 mouse BAC random-insertion transgenics, reports ∼85% endogenous-like expression for genes ≤100 kb [69], [73]. Since ∼86% of human genes are ≤100 kb (Ensemble assembly, February 2009, GRCh37/hg19), we estimate there will be suitable BACs for ∼75% of them. In addition, recombineering approaches are available to fuse two BACs, isolate only the 5′ region, or delete unwanted sequences (e.g., neighbouring genes) as needed [67]. Alternatively, the recombineering technology can be applied to a human P1 artificial chromosome should *YFG* be small [30], [66]. Our approach highlights the use of the RPCI-11 Human Male BAC Library (http://bacpac.chori.org/hmale11.htm), which was built in the pBACe3.6 vector (Figure 2A). The backbone of this BAC vector contains a *Sac*B gene that can be used as a targeting site for the first retrofitting step, adding the *HPRT* homology regions from plasmids pJDH8A/246b or the pEMS1306 series [56], [58], [74]. This BAC construct can then be used as the substrate for subsequent retrofitting steps, to add “your favourite regulatory variant” (*YFRV*), a DNA insertion, deletion, or single-base-pair alteration as needed. These retrofitting steps can be carried out in a high-throughput manner to rapidly develop an allele series of different variants to be tested. Since, both the WT-*YFG* and *YFRV*-*YFG* BAC constructs contain the homology regions that allow proper targeting at the *Hprt* locus, each can be electroporated into ESCs and selected in hypoxanthine-aminopterin-thymidine (HAT) medium, and homologous recombinant clones can be identified and microinjected into mouse embryos (Figure 2A and 2B) [63]. Male chimeras are bred to generate germline females that carry a site-specific single-copy WT-*YFG* BAC, or *YFRV*-*YFG* BAC, on their X chromosome. Assuming the genetic background is suitable, genetic complementation can be tested immediately by performing two generations of mating (Figure 2C). The resulting animals will carry a single copy of the human WT-*YFG*, or *YFRV*-*YFG* BAC, on the *Yfg* mouse null background (*Yfg* ^−/−^, *Hprt* ^WT-*YFG*^/Y or *Yfg* ^−/−^, *Hprt^YFRV^* ^-*YFG*^/Y). Animals studied on the null background will be males, thus avoiding X inactivation [56], [75]. Using this HuGX strategy, the phenotype of the *Yfg* ^−/−^, *Hprt^YFRV^* ^-*YFG*^/Y animals can be directly compared to that of the *Yfg* ^−/−^, *Hprt* ^WT-*YFG*^/Y animals. Regardless of whether interspecies complementation is complete or partial, any differences can be attributed to the function of the human variant.

**Figure 3:**
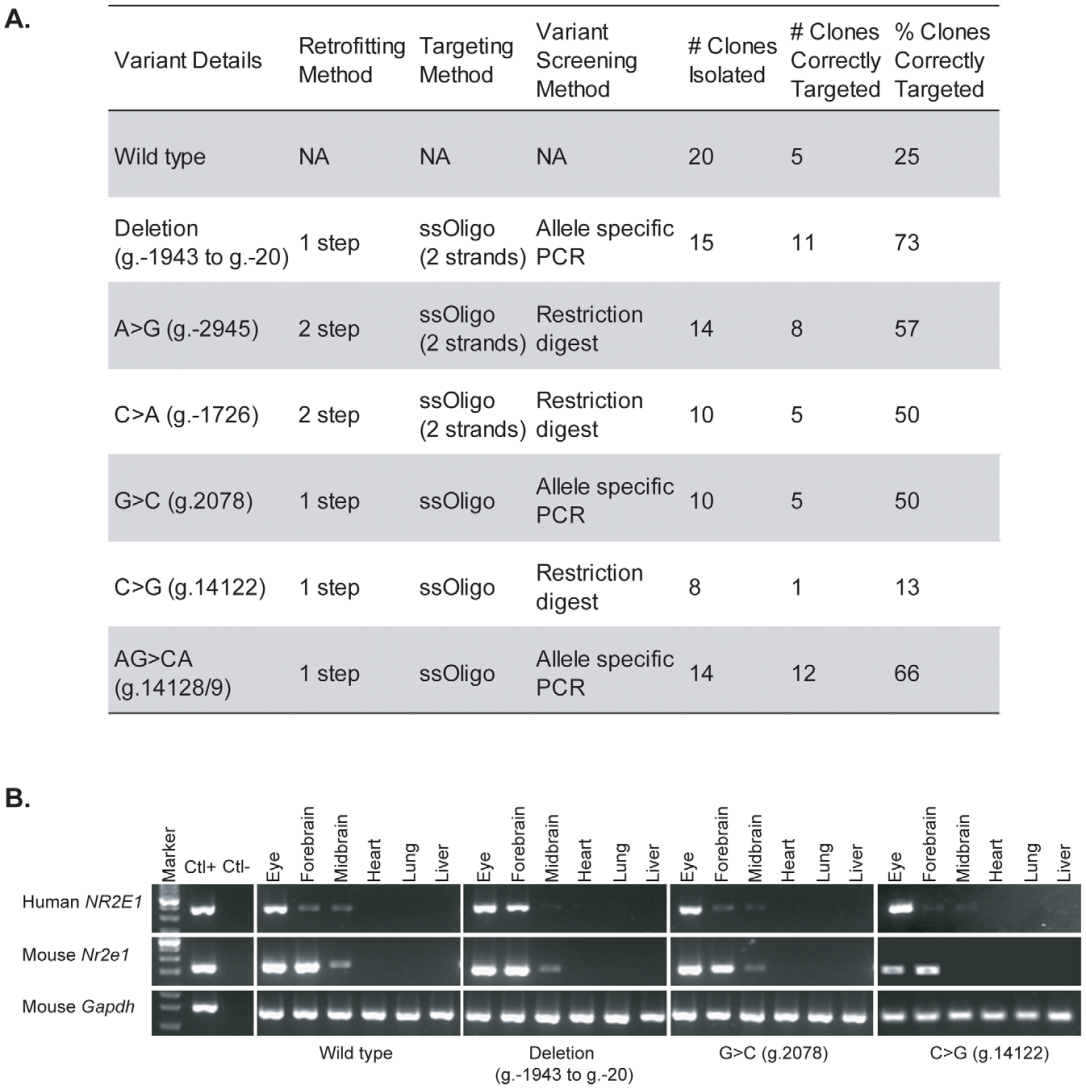
High-throughput generation of regulatory allele series. (A) Human BAC RP11-144P8 was retrofitted seven times to generate the different regulatory variants (column 1). The method of retrofitting (column 2), targeting (column 3), and variant screening (column 4) is presented for each variant. Also given are the number of ESC clones isolated after electroporation (column 5), the number of correctly targeted clones after PCR validation using assays an average of 6 kb, and a maximum of 11 kb, apart (column 6), and the resulting percentage of correctly targeted clones (column 7). (B) Species-specific reverse transcriptase PCR demonstrates transcription from the human BAC in germline animals from four of the strains generated by the high-throughput approach. One-step reverse transcription PCR reactions were performed using oligonucleotides specific for human *NR2E1*, mouse *Nr2e1*, and mouse *Gapdh*. The results show, as expected, expression of the human *NR2E1* gene in adult eye, forebrain, and midbrain, but not in adult lung, heart, and liver. Marker, 100-bp ladder; positive control (Ctl+), human RNA for human *NR2E1* assay and mouse RNA for mouse *Nr2e1* and *Gapdh* assays; negative control (Ctl−), human RNA for mouse *Nr2e1* and *Gapdh* assays and mouse RNA for human *NR2E1* assay.

We have recently used this approach to generate a directly comparable allele series for our favourite gene, *nuclear receptor 2E1* (*NR2E1*). This gene encodes an orphan nuclear receptor (also known as *TLX*) that is highly conserved between human and mouse, and has an important role in the maintenance of the neural/progenitor stem cell populations of both the forebrain and retina [76]–[80]. *Nr2e1*-null mice have brain and eye abnormalities such as hyperactivity, extreme aggressive behaviours, and blindness [81]–[83]. These phenotypes can be rescued by human *NR2E1* under its endogenous promoter, thereby demonstrating the functional equivalence of the human and mouse genes in mouse [81], [84]. Recently, positive association results between *NR2E1* and bipolar disorder have been reported, along with an increase in detection of rare variants in patients [85]. The objective of our approach was to generate seven human alleles knocked in to mouse, including one harbouring a human WT BAC, an ∼2-kb regulatory deletion, four single-base-pair candidate regulatory variants, and one two-base-pair candidate regulatory variant (Figure 3A). Recombineering and targeting of these constructs at the *Hprt* locus was performed. As shown by others and our own data (Figure 3A), it is important to molecularly characterize the integrity of the BAC insertions [56]. Nevertheless, the low number of clones that needed to be picked per construct, and the high percentage of correctly targeted clones, which varied from 13% to 73% with an average of 48%, highlights the ease of this strategy and its applicability to high throughput.

Mouse strains were generated from these different constructs, and species-specific reverse transcription PCR (RT-PCR) assays on different tissue samples were performed for four of these mouse strains (WT, Deletion [g.−1943 to g.−20], G>C [g.2078], and C>G [g.14122]) (Figure 3B). These assays demonstrated expression of the human *NR2E1* BACs in the eyes, forebrain, and midbrain of adult mice, and the absence of expression in the adult heart, lung, and liver (Figure 3B). These results, when compared to the mouse endogenous *Nr2e1* expression pattern, suggest endogenous-like tissue-specific expression of the human *NR2E1* BACs in the mouse strains. Backcrossing to the appropriate background and subsequent crossing to the *Nr2e1*-null background will allow us to evaluate the importance of these variants in the development of diseases and disorders.

Overall, generation of these seven strains has demonstrated that six to nine months is necessary to generate a single HuGX mouse model. Since the components of the HuGX methodology are scalable, and applicable to a large-scale parallel approach, this strategy is suitable for high-throughput mouse model generation to study the relevance of candidate mutations.

## Challenges for the Future

The exponential growth of data in the literature coming from GWASs and HWGS requires novel high-throughput approaches to test the biological importance of the large numbers of variants being identified, particularly candidate regulatory variants. In considering experimental approaches, three challenges face our field. The first is a consideration of the balance between construct flexibility and size. Small plasmid-based constructs lead in flexibility, especially with the option of DNA synthesis, allowing the efficient generation of any desired sequence [86]. But plasmids will almost certainly fail to capture the genomic context of the regulatory variant, especially factors such as the chromatin structure. BACs are often gene-sized (holding ∼200 kb) and are relatively easily manipulated by recombineering, and so are the construct of choice for many large projects, e.g., the GENSAT project and the International Knockout Mouse Consortium [30], [68], [69]. However, some human genes can span more than one megabase (e.g., dystrophin) [87]–[89]. YACs can accommodate this size of genomic DNA, and site-specific mutagenesis can be readily performed using the homologous recombination system of the yeast [90]. However, site-specific docking of YAC constructs is beyond our current abilities and makes YACs presently unsuitable for high-throughput single-copy approaches. The second challenge is a consideration of docking sites and technology. The *Hprt* locus provides a reliable and highly efficient docking site for BAC insertion into the mouse genome. The position of this locus on the X chromosome can be an advantage, i.e., all female offspring of a carrier male are carriers, but also a disadvantage, i.e., X inactivation in females results in mosaic expression in heterozygotes; thus, in neither sex can you obtain two functional copies of the human gene. Although the *Hprt* locus has been used to dock the largest fragments yet into the genome, up to 200 kb, size is still a limiting factor for certain human genes [56]. Hence, the generation of an alternative autosomal docking site that does not disrupt a gene, and allows insertion of large DNA fragments, would be ideal in the near future. The third challenge is a consideration of the value of stem cells and in vitro differentiation to assay candidate regulatory variant function. Mouse ESCs, as already described, can be derived to carry a matched pair of human alleles that differ only by the variant. Assuming an appropriate differentiation protocol [91], [92], differences in expression in almost any cell type could be detected, and this would add to the overall understanding of the variant. However, generation of such in vitro data alone would presumably be less successful in leading to an understanding of human disease, than when accompanied by information on the in vivo phenotype of mice derived from these same cells. A species-relevant, powerful in vitro assay can be envisaged for the near future when it would be possible to derive a matched pair of human-induced pluripotent stem cells, differing only by the variant. However, it would still remain necessary to undertake an in vivo analysis using HuMM or HuGX mice to comprehensively study the variant.

We conclude by calling for a concerted effort by the genetics community, those studying human and mouse, to move forward to solve the challenge of functional analysis of human regulatory variation in human disease and disorders.

## Ethics Statement

All mice were maintained in the pathogen-free Centre for Molecular Medicine and Therapeutics animal facility on a 6 am–6 pm light cycle, 20±2°C, with 50%±5% relative humidity, and had food and water ad libitum. All procedures involving animals were in accordance with the Canadian Council on Animal Care and University of British Columbia Animal Care Committee (Protocol# A07-0435).
